# Trajectories of change and long-term outcomes in a randomised controlled trial of internet-based insomnia treatment to prevent depression – CORRIGENDUM

**DOI:** 10.1192/bjo.2025.10913

**Published:** 2025-11-17

**Authors:** Philip J. Batterham, Helen Christensen, Andrew J. Mackinnon, John A. Gosling, Frances P. Thorndike, Lee M. Ritterband, Nick Glozier, Kathleen M. Griffiths

The authors regret the inclusion of an error in the supplementary materials of the above article. In Table DS2, the column headings under PSF suicidality were inadvertently switched. The corrected version of the table is available below.

**Table DS2:** Estimated marginal means (s.e.) and observed means (s.d.) and by intervention condition over time

	PHQ-9 NS depression		GAD-7 anxiety		ISI insomnia		PSF suicidality	
	HealthWatch mean (s.e.)	SHUTi mean (s.e.)	HealthWatch mean (s.e.)	SHUTi mean (s.e.)	HealthWatch mean (s.e.)	SHUTi mean (s.e.)	HealthWatch mean (s.e.)	SHUTi mean (s.e.)
Estimated marginal mean (s.e.)								
Baseline	5.64 (0.15)	5.84 (0.15)	5.77 (0.18)	5.83 (0.18)	16.23 (0.18)	15.92 (0.18)	0.78 (0.07)	0.74 (0.07)
Intervention week 2	6.23 (0.16)	5.93 (0.19)	6.05 (0.19)	5.36 (0.22)	14.57 (0.19)	13.22 (0.22)		
Intervention week 4	5.61 (0.17)	4.95 (0.20)	5.59 (0.20)	4.55 (0.23)	13.53 (0.22)	10.15 (0.26)		
Intervention week 6	5.75 (0.18)	3.98 (0.21)	5.87 (0.20)	3.80 (0.24)	13.27 (0.23)	8.35 (0.28)		
Intervention week 8	5.53 (0.18)	3.22 (0.21)	5.70 (0.20)	3.18 (0.23)	12.91 (0.25)	7.24 (0.30)		
Post-test	4.57 (0.16)	2.87 (0.19)	5.01 (0.19)	3.21 (0.21)	13.17 (0.25)	7.35 (0.29)	0.81 (0.08)	0.51 (0.09)
6 months	4.02 (0.19)	2.92 (0.21)	4.23 (0.19)	3.15 (0.21)	12.17 (0.27)	7.74 (0.31)	0.72 (0.09)	0.55 (0.10)
12 months	3.74 (0.19)	2.89 (0.22)	4.08 (0.22)	3.00 (0.25)	11.45 (0.30)	7.70 (0.35)	0.69 (0.09)	0.42 (0.11)
18 months	4.44 (0.27)	2.55 (0.30)	4.30 (0.27)	3.07 (0.30)	11.52 (0.40)	8.02 (0.45)	1.04 (0.15)	0.70 (0.17)
	HealthWatch mean (s.d.)	SHUTi mean (s.d.)	HealthWatch mean (s.d.)	SHUTi mean (s.d.)	HealthWatch mean (s.d.)	SHUTi mean (s.d.)	HealthWatch mean (s.d.)	SHUTi mean (s.d.)
Observed mean (s.d.)								
Baseline	5.64 (3.59)	5.84 (3.82)	5.77 (4.33)	5.83 (4.36)	16.23 (4.32)	15.92 (4.18)	0.78 (1.69)	0.74 (1.71)
Intervention week 2	6.09 (3.47)	5.70 (3.76)	5.92 (4.15)	5.04 (4.22)	14.58 (4.25)	12.98 (3.90)		
Intervention week 4	5.53 (3.54)	4.51 (3.45)	5.55 (4.55)	3.88 (3.64)	13.63 (4.72)	9.64 (4.24)		
Intervention week 6	5.84 (3.98)	3.35 (3.32)	6.01 (4.71)	3.18 (3.46)	13.45 (4.92)	7.94 (4.13)		
Intervention week 8	5.45 (3.88)	2.62 (2.89)	5.61 (4.62)	2.69 (3.07)	13.22 (5.04)	6.49 (3.96)		
Post-test	4.50 (3.66)	2.49 (2.94)	4.83 (4.39)	2.69 (3.18)	13.21 (5.45)	6.88 (4.75)	0.78 (1.80)	0.51 (1.53)
6 months	3.85 (3.63)	2.57 (3.32)	3.99 (3.77)	2.71 (3.44)	11.84 (5.13)	7.26 (4.87)	0.72 (1.71)	0.55 (1.77)
12 months	3.62 (3.46)	2.31 (2.64)	3.98 (4.15)	2.45 (3.31)	11.18 (5.54)	6.83 (4.61)	0.68 (1.80)	0.28 (0.98)
18 months	4.52 (4.30)	2.01 (2.37)	4.43 (4.20)	2.53 (2.95)	11.19 (5.78)	7.70 (5.05)	1.09 (2.41)	0.45 (1.49)

PHQ-9NS: Patient Health Questionnaire-9 without sleep item; GAD-7: Generalized Anxiety Disorder-7; ISI: Insomnia Severity Index; PSF: Psychiatric Symptom Frequency scale.

The authors apologise for this error.
